# Green supply chain decision of discarded drugs recycling: Evolutionary game and strategy simulation

**DOI:** 10.1371/journal.pone.0260235

**Published:** 2023-05-11

**Authors:** Chao Wang, Zhe Huang, Guiyu Lian

**Affiliations:** School of Business Administration, Shenyang Pharmaceutical University, Shenyang, Liaoning, China; Gonbad Kavous University, ISLAMIC REPUBLIC OF IRAN

## Abstract

The research on the recycling of discarded drugs has become an important direction of the green supply chain in China. Faced with the great challenge of recycling discarded drugs in China, it is necessary to make clear the behavior choice of each recycling subject. This paper constructs an evolutionary game model among government, drugs recycling companies and consumers, describes the evolutionary game process, and analyzes the influence of the gain of government, drug recycling enterprises and consumers on the evolutionary stable strategy trend. By using Matlab simulation software to simulate the evolution of tripartite behavior, the final result is obtained: The government’s choice of a strong regulatory strategy has an important positive role in guiding the entire discarded drug recovery market. Consumer’s choice of active participation strategy and enterprise’s choice of active participation strategy are most beneficial to the healthy development of the discarded drug recovery market. This study provides some valuable theoretical support and reference for the national decision-making of discarded drug recovery, helps to solve the problem of discarded drug recovery, and provides theoretical support and policy-making recommendations for the ultimate achievement of sustainable development goals.

## 1. Introduction

Discarded drugs are defined as the consequence of an inappropriate disposal of unused or partially used ampoules, vials, or syringes of drugs, etc. [1]. According to the National Hazardous Waste List of China [2], discarded drug produced during production, sales and use is classified as hazardous waste. Different from general waste, discarded drug is more harmful. The random discharge and storage of hazardous waste will harm the environment and human body. Now, hospitals, clinics and pharmacies give patients whole boxes of drugs. Doctors seldom fill their patients’ prescriptions on a daily basis. People have family medicine chest in their home to store commonly used drugs. Those practices ensure the convenience of drug use, but cause waste of drugs. The family will accumulate a lot of expired drugs and invalid drugs after a period of time. The research on the recycling of discarded drugs is to solve this problem [3]. In recent years, China has taken further actions on the recycling of discarded drugs. At the national level, 10 departments jointly issued the work plan for the integrated management of waste in medical institutions, calling for strict management of medical waste. The departments mainly include the National Health Commission, the National Development and Reform Commission, the Ministry of Ecology and Environment, etc. At the enterprise level, many pharmaceutical companies set an example. For example, Guangzhou Baiyunshan Pharmaceutical Co., Ltd. took the initiative to take responsibility for recovery and received a good response. Some results have been achieved in the recycling of discarded drugs, but the popularization rate of knowledge related to the identification, harm, treatment and recycling of discarded drugs is low. The harm and importance of the recycling of discarded drugs have not been paid much attention.

### 1.1. Question arise

Many problems need to be solved in the whole supply chain of discarded drug recycling. This paper wants to solve the following problems: What role should government play in the whole supply chain of discarded drug recycling, How to improve the enthusiasm of consumers and drug recycling companies for discarded drug recycling? How do government actions affect recycling businesses and consumers? How to improve the recycling efficiency of discarded drugs? How to provide some valuable theoretical support and reference for the decision-making of discarded drug recycling activities? Therefore, these issues are of theoretical importance as well as for practical significance. The paper is undertaken to address these issues. These problems not only have theoretical significance, but also practical significance. This paper aims to solve these problems.

The remainder of this paper is structured as follows. Section 2 provides a review on the existing researches. In Section 3, we introduce the tripartite game model and the basic background information, make several fundamental assumptions. Section 4 constructs a reliable model. Then in Section 5, the behavior selection of the game subject is analyzed. In section 6, the above analysis is simulated numerically. Finally, sections 7 provide managerial insights and the conclusions, the study limitations, and future research directions.

## 2. Literature review

The relevant literatures mainly focus on the following aspects: (1) Research on recycling in different fields. (2) Evolutionary game theory and its application.

### 2.1. Research on recycling in different fields

In recent years, With the continuous improvement of the level of economic development, people produce more and more kinds of wastes in production and life, waste recycling research has become the forefront of scholars’ research. When scholars do research in the analytical subfield, the most widely studied research is in the field of recycling waste electronic products. The main research direction is focused on the behavior selection of recycling subjects, while the research difference lies in the choice of research methods. Wang Wenbin et al. studied the supply chain game model [4–7], Hu Shu et al. used spider web model for subject selection analysis [8], Cao J et al. used extended producer responsibility system to study subject behavior [9]. In recent years, with the continuous promotion of waste classification policy in China, other waste products and waste recycling have gradually become a popular choice for scholars to study. The main purpose of scholars’research on waste products is to promote the progress of national policies, to promote the perfect implementation of waste classification, so scholars will choose more advanced way of research. Fei Wei et al. introduced optimization model for analysis [10], Sun Dongshi et al. introduced blockchain technology to continuously explore and promote garbage classification [11], Long Hongyu et al. introduced performance model to study the recovery of construction waste [12]. From a macro point of view, most of the research on waste recycling focuses on the supply chain. Michael Feit et al. designed a two-stage framework of recycling supply chain with fuzzy reasoning method, optimized the scene construction part and optimized the recycling mode [13]. From the perspective of reverse supply chain, many scholars have explored garbage recycling channels, recycling methods and optimal selection decisions to ensure the healthy development of garbage recycling market [3, 14–19]. In addition, starting from specific supply chain entities, such as government subsidies, producers’ environmental awareness, recyclers’ sense of responsibility, etc., Zhao Yan et al. explored the impact of these entities’ behaviors on the recycling market [20–27]. The particularity of waste medicine and the difficulty of research make the theory and application research of discarded drug recycling less. However, the above research on the theory and analysis of waste recovery has laid a solid foundation for the research of discarded drug recovery.

### 2.2. Evolutionary game theory and its application

In recent years, the theoretical research and the applied research of evolutionary game theory in economics have become one of the most popular research fields in Chinese and foreign academic circles [28]. In evolutionary game studies, the assumption of complete rationality is abandoned. The adjustment process of group behavior is regarded as a dynamic system from the perspective of system theory, which can more truly reflect the diversity and complexity of the behavior subject. It can provide theoretical basis for macro-control of group behavior [29, 30]. It can be predicted that evolutionary game theory will remain one of the most promising frontier fields in economic research for a long time to come. Besides economic research, it is also widely used in other fields. Evolutionary game research has also been fully utilized in the aspects of clothing, food, housing and transportation related to human life. In the study of food problems, Liu Dawei et al. constructed a tripartite evolutionary game model among retailers, manufacturers and government departments for the issue of expired food, and obtained the balanced and reasonable decision of all parties to deal with expired food [31]. In the medical field, Wu Xiaodan et al. established a doctor-patient evolutionary game model under the control of drug proportion, striving to improve doctor-patient relationship, and put forward relevant suggestions on graded diagnosis and treatment [32]. In the study of environmental quality supervision, Li Jian et al. Constructed a tripartite evolutionary game model of government regulatory authorities, third-party environmental testing institutions and pollutant discharge enterprises to study the different behavior choices of the three subjects. It is hoped that this study can provide important guidance for the government to strengthen environmental quality management and give relevant suggestions [33]. In the study of green innovation, He Jianmin et al. explored the evolutionary equilibrium point between green innovation and international cooperation behavior of transnational enterprises, and designed the optimal opportunism behavior governance mechanism based on the evolutionary game theory, so as to promote the realization of the win-win goal of international cooperation of transnational green innovation [34]. In the field of people’s travel and shopping, scholars use the evolutionary game method to study the government poverty alleviation system, the application of Internet of Things and China’s railway operation system, so as to improve people’s livelihood [35–40]. It is an inevitable trend that evolutionary game method is widely used in various fields.

The above scholars have made significant contributions to theoretical research on waste recycling and specific application research of evolutionary game, However, the theory and application of waste drug recycling are still seldom studied, and the research method is relatively single. Therefore, different from the previous static research model of "Environmental Impact of complex green supply chain management to control waste: a sustainable approach", this paper needs to consider that the game process of waste drug recycling is actually multiple and dynamic, and it is difficult for each entity to make a single and static decision. In this paper, evolutionary game theory is used to analyze the strategic choices of government, enterprises and consumers, which can more accurately reflect the research on the behavior choice and attitude change process of all parties. Therefore, this paper constructs an evolutionary game model among the government, drug recycling companies and consumers based on the idea of evolutionary game theory, describes the evolutionary game process, and analyzes the influence of relevant factors on the trend of evolutionary stability strategy. The strategy selection of each player is clarified in order to improve the enthusiasm of consumers and drug recycling companies for recycling, provide some valuable theoretical support and reference for the decision making of discarded drug recycling activities, and help solve the problem of discarded drug recycling. The comparison between this paper and other literatures will be shown in Table 1.

**Table 1:** A comparative overview of relevant literature research.

Research Orientation	Main research Contents
**All kinds of waste**	Behavior selection of subjects under different research models
**Evolutionary game**	The application of dynamic stable equilibrium theory in life
**Evolutionary stability strategy**	The optimal solution obtained in the evolution process
**This paper**	Green supply chain decision of discarded drugs recycling: evolutionary game and strategy simulation

## 3. Tripartite game model

### 3.1. Background description

Discarded drugs will harm human body and produce serious environmental pollution, so drug recycling is imperative. However, the development of drug recycling mechanism in China is not perfect. First of all, the subject of drug recycling is unclear, and it is not clear which subject should carry out the recycling. At present, the main recycling subjects are the government, drug companies and third-party recyclers. However, there is no consensus on which party should carry out the recycling, and there is a problem of shifting the blame among the parties; Secondly, the problem involved in the recycling process is the lack of motivation and sense of responsibility of the recycling subjects. In real life, discarded drugs cannot be reused due to their special properties and ethical issues. Therefore, the recycling subjects can not gain many benefits from it, so the recycling motivation is insufficient. The last problem is that Chinese residents have little understanding of the harm of discarded drugs, and do not know the severity of the harm of discarded drugs to the environment. The lack of environmental awareness leads to the lack of active recycling awareness among residents, and the phenomenon that drugs are discarded at will is serious.

### 3.2. Symbolic description

The variables and definitions used in this study are shown in Table 2.

**Table 2:** Definition of variables.

Variables	Definition	Notes
*A*1, *A*2	Incentives for active participation by firms and consumers in times of strong government regulation	
*F*_1_	Punishment for passive participation in recycling companies when the government is enforcing regulations	
*C*1, *C*2	Costs incurred when businesses and consumers actively participate in recycling	*C*_1_ ≥ *C*_3_
*C*_3_, *C*_4_	Cost of passive participation by businesses and consumers in recycling	*C*_2_ ≥ *C*_4_
*C*_5_, *C*_6_	The cost of strong and weak government regulation	*C*_5_ ≥ *C*_6_
*R*_1_, *R*_3_	The gain of active and passive participation of companies	
*R*2, *R*4	The gain of active and passive consumer participation	
*R*_5_, *R*_6_	The gain of strong government regulation and weak government regulation	
*M*1, *M*2	The losses brought to the government and consumers when companies participate passively	
*M*_3_, *M*_4_	The losses brought to the government and companies by consumers’ passive participation	
*B*1, *B*2	The incentive for companies and consumers when the government has strong supervision	

### 3.3. Model assumptions

In order to establish a reasonable three-party evolutionary game model, this paper makes the following assumptions:

#### Assumption 1

In this paper, a discarded drug recycling system composed of "government—recycler—consumer" is studied. Three game subjects have finite rationality, and the information between the subjects is asymmetric, and the behavior of the game subjects is random.

#### Assumption 2

Behavior strategy selection of game subject:

Strategies of government: for the recycling behavior of drug recycling companies, the government can choose two strategies: strong supervision or weak supervision. The entire process of recycling discarded drugs cannot be done without government support and supervision. However, the government can choose strong supervision or weak supervision. The main reason is that strong supervision will increase the cost of government, so the government will not firmly choose strong supervision. The government should provide policy support, technical support, equipment investment and other measures for recycling companies to encourage drug recycling companies to actively recycle discarded drugs, and guide drug recycling companies to enhance their sense of responsibility. The government regulates the recycling practices of drug recycling companies and provides appropriate support and subsidies to responsible companies. On the contrary, companies that lack responsibility consciousness and treat drug recycling negatively will be punished to some extent. The government should give some encouragement to consumers who are more environmentally conscious and actively participate in the recycling of discarded drug.

Strategies of recycling companies: recycling companies can choose active recycling strategies when they are conscious of their responsibilities, which will help to recycle discarded drugs, reduce environmental pollution, and improve the ecological environment. When recycling companies are not conscious of their responsibilities, they may choose negative recycling strategies, which do not have a positive effect on the environment. When the government chooses strong supervision and companies choose to actively participate in drug recycling, both of them can improve the recycling rate of discarded drugs, thus reducing environmental pollution and other problems caused by discarded drugs. Meanwhile, they can also improve the reputation and goodwill of recycling companies in the whole industry and consumers.

Strategies of consumers: Consumers will choose to actively participate in the recycling strategy when they are more environmentally conscious, and they will choose a negative participation strategy when they are less environmentally conscious.

#### Assumption 3

When the government chooses weak regulation, in other wordsgain, when the constraint condition *R*_1_ –*C*_1_ ≤ *R*_3_ –*C*_3_, *R*_2_ –*C*_2_ ≤ *R*_4_ –*C*_4_ is satisfied, both consumers and companies will behave opportunistically. When the constraint condition *R*_3_ –*C*_3_ –*F*_1_ ≤ *R*_1_ –*C*_1_ + *A*_1_, *R*_4_ –*C*_4_ ≤ *R*_2_ –*C*_2_ + *A*_2_ is satisfied, strong government regulation will have a certain restrictive effect on the behavior of companies and consumers.

#### Assumption 4

This paper defines that the government rewards, subsidizes, or penalizes companies and consumers only when it chooses a strong regulatory strategy, and does not take action against companies and consumers when it chooses a weak regulatory strategy.

#### Assumption 5

The difference of the gain from the government’s strong regulatory approach minus the gain from the government’s weak regulation is defined as the government’s relative gain.

## 4. Model construction

### 4.1. Payment matrix construction

Specifically, it is assumed that the probability of the government to conduct strong supervision and weak supervision is *x* and (1 –*x*), when the sense of responsibility of drug recycling companies is strong, the probability of actively recycling waste drugs is *y*, and the probability of negative recycling of waste drugs is (1 –*y*), when the sense of responsibility is weak. The probability of positive participation in drug recycling was *z* when consumers have strong environmental awareness, and (1 –*z*) was is the probability of negative participation in drug recycling when environmental awareness was weak. (*x*, *y*, *z*) reflects the evolutionary dynamics of the government, drug recycling companies and consumers in the recycling process of discarded drugs.

### 4.2. Replication dynamic equation

The actions of the government, recycling companies, and consumers will interact and influence each other. Groups of strategies with lower gain will imitate groups of strategies with higher gain and shift to them. In the following, the formation conditions and process of evolutionary stability strategy are obtained by establishing the replication dynamic equation of three-party evolutionary game. Specifically, In this paper, the expected gain and overall gain of the government, recycling enterprises and consumers when they adopt different strategies are calculated respectively.

(1) According to the game matrix of the government, recycling companies and consumers in Table 3, it can be concluded that the expected gain of the government’s selection of strong supervision is:

$$\begin{array}{l} E_{x}=yz(R_{5}-C_{5}-A_{1}-B_{1}-A_{2}-B_{2})\\ \;+y(1-z)(R_{5}-C_{5}-A_{1}-B_{1}-B_{2}-M_{3})\\ \;+(1-y)z(R_{5}-C_{5}+F_{1}-B_{1}-A_{2}-B_{2}-M_{1})\\ \;+(1-y)(1-z)(R_{5}-C_{5}-B_{1}-B_{2}-M_{1}-M_{3}+F_{1}) \end{array}$$ (1)

**Table 3:** Payment matrix of government, recycling companies and consumers.

		Recycling companies	consumers	
			*z*	1 –*z*
**The government**	*x*	*y*	*R*_5_ –*C*_5_ –*A*_1_ –*B*_1_ –*A*_2_ –*B*_2_ *R*_1_ –*C*_1_ + *A*_1_ + *B*_1_ *R*_2_ –*C*_2_ + *A*_2_ + *B*_2_	*R*_5_ –*C*_5_ –*A*_1_ +*B*_1_ –*B*_2_ –*M*_3_ *R*_1_ –*C*_1_ + *A*_1_ –*B*_1_ –*M*_4_ *R*_4_ –*C*_4_ + *B*_2_
		1 –*y*	*R*_5_ –*C*_5_ + *F*_1_ –*B*_1_ –*A*_2_ –*B*_*2*_ *–M*_1_ *R*_3_ –*C*_3_ + *B*_1_ –*F*_1_ *R*_2_ –*C*_2_ + *A*_2_ + *B*_2_ –*M*_2_	*R*_5_ –*C*_5_ –*B*_1_ –*B*_2_ –*M*_1_ –*M*_3_ + *F*_1_ *R*_3_ –*C*_3_ + *B*_1_ –*F*_1_ –*M*_4_ *R*_4_ –*C*_4_ + *B*_2_ –*M*_2_
	1 –*x*	*y*	*R*_6_ –*C*_6_ *R*_1_ –*C*_1_ *R*_2_ –*C*_2_	*R*_6_ –*C*_6_ + *M*_3_ *R*_1_ –*C*_1_ –*M*_4_ *R*_4_ –*C*_4_
		1 –*y*	*R*_6_ –*C*_6_ –*M*_1_ *R*_3_ –*C*_3_ *R*_2_ –*C*_2_ –*M*_2_	*R*_6_ –*C*_6_ –*M*_1_ –*M*_3_ *R*_3_ –*C*_3_ –*M*_4_ *R*_4_ –*C*_4_ –*M*_2_

The expected gain of the government’s selection of weak supervision is:

$$\begin{array}{l} E_{1-x}=yz(R_{6}-C_{6})\\ \;+y(1-z)(R_{6}-C_{6}-M_{3})\\ \;+(1-y)z(R_{6}-C_{6}-M_{1})\\ \;+(1-y)(1-z)(R_{6}-C_{6}-M_{1}-M_{3}) \end{array}$$ (2)

The average expected gain of the government’s mixed strategy is:

$$\bar{EG}=xE_{x}+(1-x)E_{1-x}^{{}_{}}$$ (3)

The replication dynamic equation of government strategy can be obtained as follows:

$$\begin{array}{l} \frac{d_{x}}{d_{t}}=x(E_{x}-\bar{EG)}=x(1-x)(E_{x}-E_{1-x})\\ \;=x(1-x)[-y(A_{1}+F_{1})-zA_{2}+R_{5}-C_{5}\\ \;-B_{1}-B_{2}+F_{1}-R_{6}+C_{6}] \end{array}$$ (4)

(2) the expected gain of recycling companies adopting active recycling strategies is:

$$\begin{array}{l} E_{y}=xz(R_{1}-C_{1}+A_{1}+B_{1})\\ \;+x(1-z)(R_{1}-C_{1}+A_{1}+B_{1}-M_{4})\;\\ \;+(1-x)z(R_{1}-C_{1})\\ \;+(1-x)(1-z)(R_{1}-C_{1}-M) \end{array}$$ (5)

The expected gain of recycling companies adopting negative recycling strategies is:

$$\begin{array}{l} E_{1-y}=xz(R_{3}-C_{3}+B_{1}-F_{1})\\ \;+x(1-z)(R_{3}-C_{3}+B_{1}-F_{1}-M_{4})\\ \;+(1-x)z(R_{3}-C_{3})\\ \;+(1-x)(1-z)(R_{3}-C_{3}-M_{4}) \end{array}$$ (6)

The average expected gain of recycling companies adopting mixed strategies is:

$$\bar{EW}=yE_{y}+(1-y)E_{1-y}$$ (7)

The replication dynamic equation of recycling enterprise strategy can be written:

$$\begin{array}{l} \frac{d_{y}}{d_{t}}=y(E_{y}-\bar{E_{W}})=y(1-y)(E_{y}-E_{1-y})\\ \;=y(1-y)[x(A_{1}+F_{1})+R_{1}-C_{1}-R_{3}+C_{3}] \end{array}$$ (8)

(3) The expected gain of consumers adopting active strategies is:

$$\begin{array}{l} E_{z}=xy(R_{2}-C_{2}+A_{2}+B_{2})\\ \;+x(1-y)(R_{2}-C_{2}+A_{2}+B_{2}-M_{2})\\ \;+(1-x)y(R_{2}-C_{2})\\ \;+(1-x)(1-y)(R_{2}-C_{2}-M_{2}) \end{array}$$ (9)

The expected gain of consumers adopting negative strategies is:

$$\begin{array}{l} E1-z=xy(R_{4}-C_{4}+B_{2})\\ \;+x(1-y)(R_{4}-C_{4}+B_{2}-M_{2})\\ \;+(1-x)y(R_{4}-C_{4})\\ \;+(1-x)(1-y)(R_{4}-C_{4}-M_{2}) \end{array}$$ (10)

The expected gain of consumers adopting a mixed strategy is:

$$\bar{ES}=zE_{z}+(1-z)E_{1-z}$$ (11)

The replication dynamic equation of consumer strategy can be obtained as:

$$\begin{array}{l} \frac{d_{z}}{d_{t}}=z(1-z)(E_{z}-E_{1-z})\;\\ \;=z(1-z)(xA_{2}+R_{2}-C_{2}-R_{4}+C_{4}) \end{array}$$ (12)

### 4.3. Solve for equilibrium points

The tripartite replication dynamic equation of the government, recycling companies and consumers can be obtained as follows:

$$\left\{\begin{array}{c} F(x)=x(1-x)\left[-y(A_{1}+F_{1})-zA_{2}+R_{5}-C_{5}-B_{1}-B_{2}+F_{1}-R_{6}+C_{6}\right]\\ F(y)=y(1-y)[x(A_{1}+F_{1})+R_{1}-C_{1}-R_{3}+C_{3}]\\ F(z)=z(1-z)(xA_{2}+R_{2}-C_{2}-R_{4}+C_{4}) \end{array}\right.$$ (13)

In this system, *F*(*x*) = *F*(*y*) = *F*(*z*) = 0, the equilibrium points of the evolutionary game process can be obtained as E1(0, 0, 0), E2(0, 0, 1), E3(0, 1, 0), E4(0, 1, 1), E5(1, 0), E6(1, 0, 1), E7(1, 1, 0), and E8(1, 1, 1). Because in an asymmetric game, if the evolutionary game equilibrium E is an evolutionary stable equilibrium, then E must be a strict Nash equilibrium, and the strict Nash equilibrium is a pure strategy equilibrium. in other wordsgain, the mixed strategy equilibrium in an asymmetric game must not be an evolutionary stable equilibrium [41], Therefore, we only need to discuss the asymptotic stability of pure strategy equilibrium, we only need to analyze the asymptotic stability of the Nash equilibrium points of E1-E8 in this paper.

### 4.4. Evolutionary stability strategy solution

#### 4.4.1. Stability analysis of equilibrium points

According to Friedman’s method, the evolutionary stability strategy of a differential equation system can be derived from the local stability analysis of the Jacobian matrix of the system [42], while the asymptotic stability of the equilibrium point is determined by the Lyapunov criterion (indirect method) [43]. First, the Jacobian matrix and its eigenvalues are solved, and the partial derivatives of *F*(*x*), *F*(*y*), *F*(*z*) with respect to *x*, *y*, *z* are obtained respectively. Then, the Jacobian matrix of this system is as follows:

$$\left[\begin{array}{ccc} \begin{array}{l} (1-2x)[-y(A_{1}+F_{1})-zA_{2}+R_{5}\\ -C_{5}-B_{1}-B_{2}+F_{1}-R_{6}+C_{6}] \end{array} & x(1-x)(A_{1}+F_{1}) & x(x-1)A_{2}\\ y(1-y)(A_{1}+F_{1}) & \begin{array}{l} (1-2y)[x(A_{1}+F_{1})+R_{1}\\ -C_{1}-R_{3}+C_{3}] \end{array} & 0\\ z(1-z)A_{2} & 0 & \begin{array}{l} (1-2z)(xA_{2}+R_{2}\\ -C_{2}-R_{4}+C_{4}) \end{array} \end{array}\right]$$ (14)

The Jacobian matrix of pure strategy Nash equilibrium point E1(0, 0, 0) is:

$$\left[\begin{array}{ccc} R_{5}-C_{5}-B_{1}-B_{2}+F_{1}-R_{6}+C_{6} & 0 & 0\\ 0 & R_{1}-C_{1}-R_{3}+C_{3} & 0\\ 0 & 0 & R_{2}-C_{2}-R_{4}+C_{4} \end{array}\right]$$ (15)

At this time, the eigenvalue of the Jacobian matrix can be written:

$$\begin{array}{l} \lambda_{1}=R_{5}-C_{5}-B_{1}-B_{2}+F_{1}-R_{6}+C_{6},\\ \lambda_{2}=R_{1}-C_{1}-R_{3}+C_{3},\\ \lambda_{3}=R_{2}^{}-C_{2}-R_{4}+C_{4} \end{array}$$ (16)

According to the above model assumptions, the relationship between the eigenvalue of the equilibrium point E1(0, 0, 0) and 0 is *λ*_2_ < 0, *λ*_3_ < 0 According to the Lyapunov discriminant method (indirect method), when *λ*_1_ < 0, E1(0, 0, 0) is asymptotically stable. When *λ*_1_ > 0, E1(0, 0, 0) is the saddle point. The stability proof procedure of other Nash equilibrium points is similar to the above. As mentioned above, stability analysis of the 8 pure strategy equilibrium points is shown in Table 4, and then the following theorem is obtained.

**Table 4:** Stability analysis of equilibrium points.

Equilibrium	The eigenvalue	Stability analysis
E1(0,0,0)	*λ*_1_ *= R*_5_ –*C*_5_ –*B*_1_ –*B*_2_ + *F*_1_ –*R*_6_ + *C*_6_ *λ*_2_ *= R*_1_ –*C*_1_ –*R*_3_ + *C*_3_ < 0 *λ*_3_ *= R*_2_ –*C*_2_ –*R*_4_ + *C*_4_ < 0	When *λ*_1_ < 0, E1 is asymptotically stable. When *λ*1 > 0, E1 is the saddle point.
E2(0,0,1)	*λ*_1_ *= –A*_2_ + *R*_5_ –*C*_5_ –*B*_1_ –*B*_2_ + *F*_1_ –*R*_6_ + *C*_6_ *λ*_2_ *= R*_1_ –*C*_1_ –*R*_3_ + *C*_3_ < 0 *λ*_3_ *= –*(*R*_2_ –*C*_2_ –*R*_4_ + *C*_4_) > 0	E2 is a saddle point.
E3(0,1,0)	*λ*_1_ *= –*(*A*_1_ + *F*_1_) + *R*_5_ + *C*_5_ –*B*_1_ –*B*_2_ + *F*_1_ –*R*_6_ + *C*_6_ *λ*_2_ *= –*(*R*_1_ –*C*_1_ –*R*_3_ + *C*_3_) > 0 *λ*_3_ *= R*_2_ –*C*_2_ –*R*_4_ + *C*_4_) < 0	E3 is a saddle point.
E4(0,1,1)	*λ*_1_ *= –*(*A*_1_ + *F*_1_)–*A*_2_ + *R*_5_ –*C*_5_ –*B*_1_ –*B*_2_ + *F*_1_ –*R*_6_ + *C*_6_ *λ*_2_ *= –*(*R*_1_ –*C*_1_)–*R*_3_ + *C*_3_ > 0 *λ*_3_ *= –*(*R*_2_ –*C*_2_ –*R*_4_ + *C*_4_) > 0	When *λ*1 > 0, E4 is the unstable point. When *λ*1 < 0, E4 is the saddle point.
E5(1,0,0)	*λ*_1_ *= –*(*R*_5_ –*C*_5_ –*B*_1_ –*B*_2_ + *F*_1_ –*R*_6_ + *C*_6_) *λ*_2_ *= A*_1_ + *F*_1_ + *R*_1_ –*C*_1_ –*R*_3_ + *C*_3_ > 0 *λ*_3_ *= A*_2_ + *R*_2_ –*C*_2_ –*R*_4_ + *C*_4_ > 0	When *λ*1 > 0, E5 is the unstable point. When *λ*1 < 0, E5 is the saddle point.
E6(1,0,1)	*λ*_1_ *= –*(*–A*_2_ + *R*_5_ –*C*_5_ –*B*_1_ –*B*_2_ + *F*_1_ –*R*_6_ + *C*_6_) *λ*_2_ *= A*_1_ + *F*_1_ + *R*_1_ –*C*_1_ –*R*_3_ + *C*_3_ > 0 *λ*_3_ *= –*(*A*_2_ + *R*_2_ –*C*_2_ –*R*_4_ + *C*_4_) < 0	E6 is a saddle point
E7(1,1,0)	*λ*_1_ *= –*[–(*A*_1_ + *F*_1_) + *R*_5_ –*C*_5_ –*B*_1_ –*B*_2_ + *F*_1_ –*R*_6_ + *C*_6_] *λ*_2_ *= –*(*A*_1_ + *F*_1_ + *R*_1_ –*C*_1_ –*R*_3_ + *C*_3_) < 0 *λ*_3_ *= A*_2_ + *R*_2_ –*C*_2_ –*R*_4_ + *C*_4_) > 0	E7 is a saddle point.
E8(1,1,1)	*λ*_1_ *= –*[–(*A*_1_ + *F*_1_)–*A*_2_ + *R*_5_ –*C*_5_ –*B*_1_ –*B*_2_ + *F*_1_ –*R*_6_ + *C*_6_] *λ*_2_ *= –*(*A*_1_ + *F*_1_ + *R*_1_ –*C*_1_ –*R*_3_ + *C*_3_) < 0 *λ*_3_ *= –*(*A*_2_ + *R*_2_ –*C*_2_ –*R*_4_ + *C*_4_) < 0	When *λ*1 < 0, E8 is asymptotically stable, and when *λ*1 > 0, E8 is saddle point.

#### 4.4.2. Evolutionary stability strategy analysis

The following theorems can be obtained from the above equilibrium stability table:

Theorem 1: When the constraint condition–(*A*_1_ + *F*_1_)–*A*_2_ + *R*_5_ –*C*_5_ –*B*_1_ –*B*_2_ *+F*_1_ –*R*_6_ + *C*_6_ > 0 is equivalent to *R*_5_ –*R*_6_ > *C*_5_ –*C*_6_ + *B*_1_ *+B*_2_ + *A*_1_ +*A*_2_, E8(1, 1, 1) is the only asymptotically stable point.

**Fig 1:**
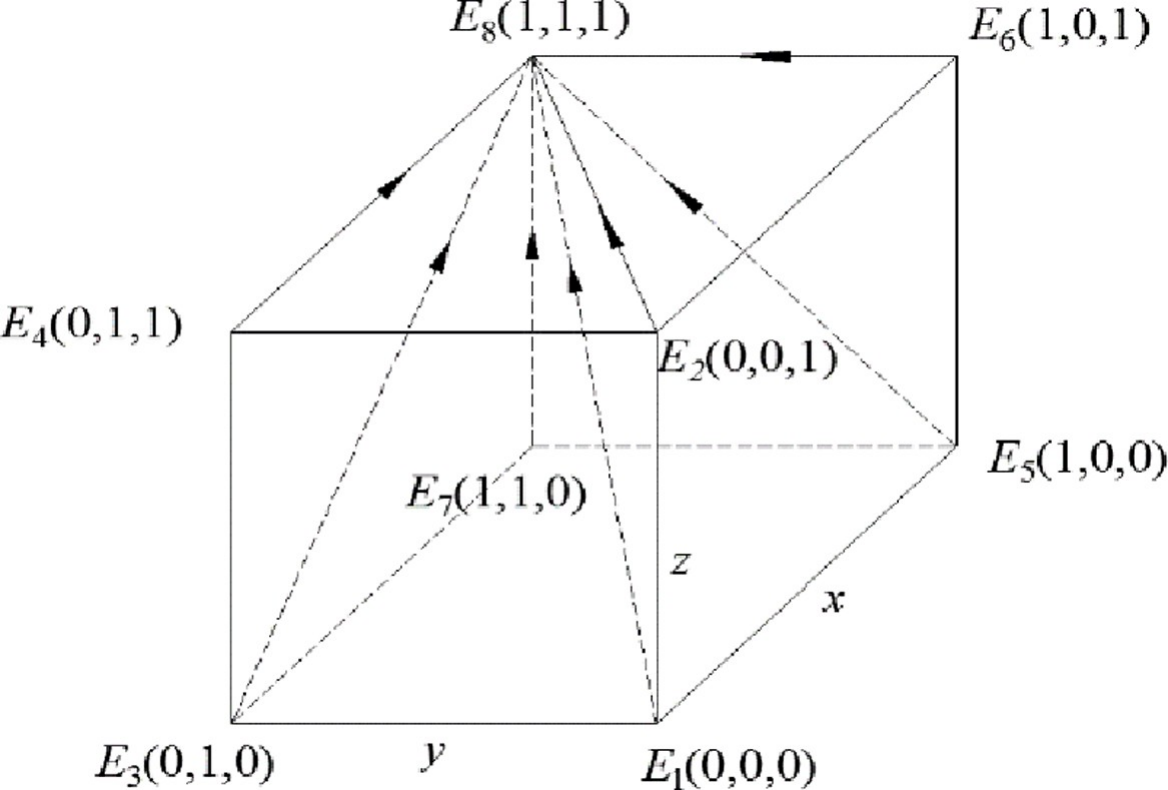
Phase diagram when the constraint condition is *R*_5_ –*R*_6_ > *C*_5_ –*C*_6_ + *B*_1_ *+B*_2_ + *A*_1_ +*A*_2_.

Theorem 1 shows that the government will choose the strategy of strong regulation when the gain difference between strong regulation and weak regulation is large, in other wordsgain, the government will choose the strategy of strong regulation when the gain of strong regulation is larger than that of weak regulation. Under this condition, recycling companies will choose the most favorable strategy to actively participate in recycling, and consumers will choose to actively participate in recycling due to strong government supervision. In this case, the dynamic system composed of the government, the discarded drug recycling companies and consumers is stable in the strategy combination (strong supervision, active participation, active participation), and the entire discarded drug recycling system is in the most environmentally friendly state. The phase diagram of this model is shown in Fig 1.

Theorem 2: when the constraint condition *R*_5_ –*C*_5_ –*B*_1_ –*B*_2_ + *F*_1_ –*R*_6_ + *C*_6_ < 0 is equivalent to *R*_5_ –*R*_6_ < *C*_5_ –*C*_6_ + *B*_1_ + *B*_2_ –*F*_1_, E1(0, 0, 0) is the only asymptotically stable point.

**Fig 2:**
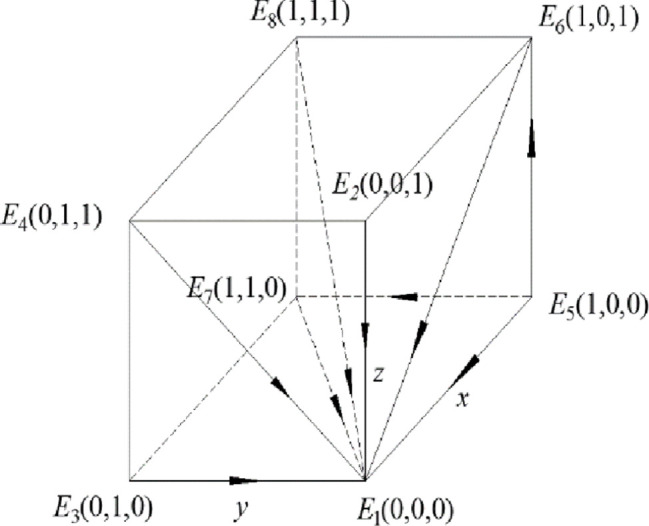
Phase diagram when the constraint condition is *R*_5_ –*R*_6_ < *C*_5_ –*C*_6_ + *B*_1_ + *B*_2_ –*F*_1_.

Theorem 2 shows that when the gain difference between the gain of strong regulation and weak regulation is small, in other words, when the gain of strong regulation of recycling companies and consumers are smaller than the gain of weak regulation, the enthusiasm of government regulation is discouraged, the government will choose a weak regulatory strategy. Under this condition, discarded drug recycling companies lack the sense of responsibility for recycling and are unwilling to spend a lot of human and material resources due to the existence of opportunistic behaviors. In order to maximize their own gain, recycling companies will choose the negative participation strategy. At this time, consumers also choose the negative participation strategy due to the lack of environmental awareness and the pursuit of maximizing their own interests. The entire discarded drug recycling environment is poor, which will cause great harm to the environment. In this case, the dynamic system composed of the government, the discarded drug recycling enterprise and the consumer is stable in the strategy combination (weak regulation, negative participation, negative participation), and neither party has the motivation to change its behavior, so the whole dynamic system is in a chaotic and stable state. The phase diagram of this model is shown in Fig 2.

## 5. Analysis of game subject evolution

Through the analysis of the above-mentioned evolutionary stable strategy of the game players, it can be seen that no matter what strategy the government chooses to adopt, the situation that the waste medicine recycling companies choose to participate passively and consumers choose to participate passively will also exist for a long time. in other wordsgain, recycling companies and consumers choose (passive participation, passive participation), (active participation, active participation) strategy will co-exist for a long time. The evolutionary stable convergence trend of an evolutionary game system is influenced by the payment matrix parameters and converges to different equilibrium points. Therefore, this paper will analyze in depth the impact of different factors on the stabilization strategy of the evolutionary game, which are the gain differential between strong and weak government regulation, the cost of opportunistic behavior of recycling companies and consumers, and the regulatory intensity of strong and weak government regulation. The evolutionary stability analysis of this paper is based on the government’s selection of different regulatory strategies, when the gain and costs of consumer opportunism meet the condition *R*_1_ –*C*_1_ ≤ *R*_3_ –*C*_3_, *R*_2_ –*C*_2_ ≤ *R*_4_ –*C*_4_ and *R*_3_ –*C*_3_ –*F*_1_ ≤ *R*_1_ –*C*_1_ + *A*_1_, *R*_4_ –*C*_4_ ≤ *R*_2_ –*C*_2_ + *A*_2_, when the above conditions change, the evolutionary stability strategy of equilibrium point will also change accordingly. Therefore, the following content will focus on analyzing the evolution and stability strategies of game players when the above conditions change.

### 5.1. The change of relative government gain

One of the important conditions for the above evolutionary stability strategy analysis is that the stable equilibrium point can be obtained when the relative gain of the government is relatively high, in other wordsgain, when the restriction condition *R*_5_ –*R*_6_ > *C*_5_ –*C*_6_ + *B*_1_ + *B*_2_ + *A*_1_ + *A*_2_ is satisfied, or when the relative gain of the government is low, in other wordsgain, when the restriction condition *R*_5_ –*R*_6_ < *C*_5_ –*C*_6_ + *B*_1_ + *B*_2_ –*F*_1_ is satisfied. Therefore, *C*_5_ –*C*_6_ + *B*_1_ + *B*_2_ –*F*_1_ < *R*_5_ –*R*_6_ *< C*_5_ –*C*_6_ + *B*_1_ + *B*_2_ + *A*_1_ + *A*_2_ is considered below, in other wordsgain, when the government’s relative gain is between high and low, the evolutionary game stability strategy is shown in the table below.

It can be seen from Table 5 that when the government’s relative gain is between high and low, the dynamic system composed of the government, discarded drug recycling companies and consumers is in an unstable state, the system has no evolutionarily stable point.

**Table 5:** Stability analysis of the equilibrium point at *C*_5_ –*C*_6_ + *B*_1_ + *B*_2_ –*F*_1_ < *R*_5_ –*R*_6_ *< C*_5_ –*C*_6_ + *B*_1_ + *B*_2_ + *A*_1_ + *A*_2_.

Equilibrium		E1	E2	E3	E4	E5	E6	E7	E8
**The eigenvalue**	*λ*1	> 0	-	-	> 0	< 0	-	-	> 0
	*λ*2	< 0	< 0	> 0	> 0	> 0	> 0	< 0	< 0
	*λ*3	< 0	> 0	< 0	> 0	> 0	< 0	> 0	< 0
**Stability analysis**		Saddle point	Saddle point	Saddle point	Instability point	Saddle point	Saddle point	Saddle point	Saddle point

### 5.2. Opportunistic behaviors of companies and consumers

It is pointed out in the hypothesis of this paper that when the government chooses weak regulation, both consumers and companies will have opportunistic behaviors. Therefore, this paper will analyze the situation when the cost of opportunism is high, in other wordsgain, when the cost for discarded drug recycling companies and consumers to choose negative participation is high, namely *R*_1_ –*C*_1_ ≥ *R*_3_ –*C*_3_, *R*_2_ –*C*_2_ ≥ *R*_4_ –*C*_4_. At this point, the situation of the evolutionary stable equilibrium point is shown in the following table.

According to Table 6 and Theorem 1 and 2, theorem 3 can be obtained. When *R*_5_ –*R*_6_ < *C*_5_ –*C*_6_ + *B*_1_ + *B*_2_ + *A*_1_ + *A*_2_, the evolutionary stable point E4 (0, 1, 1) is the only evolutionary stable point, and when *R*_5_ –*R*_6_ > *C*_5_ –*C*_6_ + *B*_1_ + *B*_2_ + *A*_1_ + *A*_2_, the evolutionary stable point E8(1, 1, 1) is the only evolutionary stable point.

**Table 6:** Stability analysis of equilibrium point at *R*_1_ –*C*_1_ ≥ *R*_3_ –*C*_3_, *R*_2_ –*C*_2_ ≥ *R*_4_ –*C*_4_.

equilibrium		E1	E2	E3	E4	E5	E6	E7	E8
The eigenvalue	*λ*1		-	-		-	-	-	
	*λ*2	> 0	> 0	> 0	< 0	> 0	> 0	< 0	< 0
	*λ*3	> 0	< 0	< 0	< 0	< 0	< 0	> 0	< 0
Stability analysis		When *λ*1 > 0, E1 is the unstable point; when *λ*1 < 0, E1 is the saddle point	Saddle point	Saddle point	When *λ*1 > 0, E4 is the saddle point; When *λ*1 < 0, E4 is the stable point	Saddle point	Saddle point	Saddle point	When *λ*1 > 0, E8 is the saddle point; When *λ*1 < 0, E8 is the stable point

Theorem 3 shows that when the opportunity cost of companies and consumers is high, no matter whether the government chooses strong supervision or weak supervision, discarded drug recycling companies and consumers will steadily choose to actively participate in the behavior after a long evolution process. Therefore, when it’s relative gain is low, (weak supervision, active participation, active participation) is the only stable equilibrium; on the contrary, if the government’s relative revenue is high, the only stable strategy of the tripartite evolutionary game composed of government, enterprise and consumer is (strong supervision, active participation, active participation).

**Fig 3:**
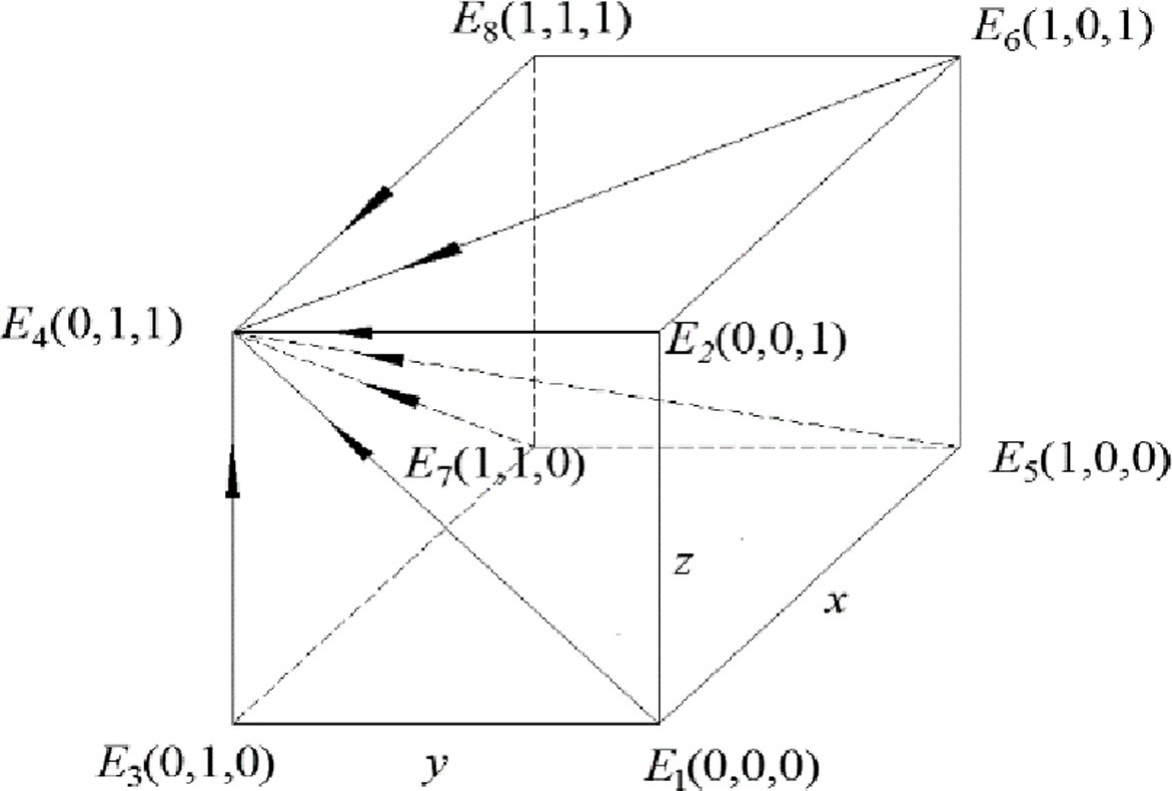
Phase diagram when the constraint condition is *R*_5_ –*R*_6_ < *C*_5_ –*C*_6_ + *B*_1_ + *B*_2_ + *A*_1_ + *A*_2_.

**Fig 4:**
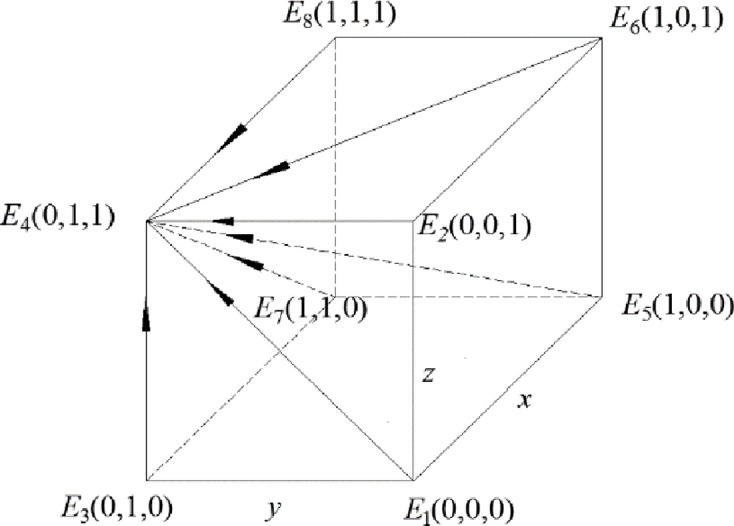
Phase diagram when the constraint condition is *R*_5_ –*R*_6_ > *C*_5_ –*C*_6_ + *B*_1_ + *B*_2_ + *A*_1_ + *A*_2_.

The phase diagrams of this model are shown in Figs 3 and 4.

### 5.3. Changes in the intensity of government supervision

In this part, the paper will analyze the stability of each equilibrium point when the government adopts a strong regulatory strategy, but the regulatory intensity is still insufficient, and the opportunistic behavior of recycling companies and consumers cannot be changed. The stability of the evolutionary equilibrium point is analyzed at *R*_3_ –*C*_3_ –*F*_1_ > *R*_1_ –*C*_1_ + *A*_1_, *R*_4_ –*C*_4_ > *R*_2_ –*C*_2_ + *A*_2_.

The evolutionary stability analysis of the equilibrium point is shown in Table 7.

**Table 7:** Stability analysis of equilibrium points at *R*_3_ –*C*_3_ –*F*_1_ > *R*_1_ –*C*_1_ + *A*_1_, *R*_4_ –*C*_4_ > *R*_2_ –*C*_2_ + *A*_2_.

Equilibrium		E1	E2	E3	E4	E5	E6	E7	E8
**The eigenvalue**	*λ*1		-	-			-	-	
	*λ*2	< 0	> 0	> 0	> 0	< 0	< 0	> 0	> 0
	*λ*3	< 0	< 0	< 0	> 0	< 0	> 0	< 0	> 0
**Stability analysis**		When *λ*1 > 0, E1 is the saddle point. When *λ*1 < 0, E1 is the stable point	Saddle point	Saddle point	When *λ*1 > 0, E4 is the unstable point when *λ*1 < 0, E4 is the saddle point	When *λ*1 > 0, E5 is the saddle point; When *λ*1 < 0, E5 is the stable point	Saddle point	Saddle point	When *λ*1 > 0, E8 is the unstable point when *λ*1 < 0, E8 is the saddle point

Theorem 4 can be obtained from Table 6, When the restriction condition *R*_5_ –*R*_6_ < *C*_5_ –*C*_6_ + *B*_1_ + *B*_2_ –*F*_1_ is satisfied, E1 (0, 0, 0) is the only evolutionary equilibrium point, and when the restriction condition *R*_5_ –*R*_6_ > *C*_5_ –*C*_6_ + *B*_1_ + *B*_2_ –*F*_1_ is satisfied, E5 (1, 0) is the only evolutionary equilibrium point.

Theorem 4 shows that when the government supervision is still not strong enough, discarded drug recycling companies and consumers will steadily jointly choose the recycling strategy of negative participation after a long evolution. Therefore, when the relative gain of government regulation is low, it will lead to the lack of enthusiasm of government regulation and the government will choose weak regulation. The only stable strategy combination of this system is (weak regulation, passive participation, passive participation). On the contrary, when the relative gain of government regulation is relatively high, the government regulation enthusiasm is high, and the only stable strategy combination of the system is (strong regulation, negative participation, negative participation). In the whole recycling process of discarded drugs, when the government has insufficient supervision, whether the government chooses strong supervision or weak supervision, recycling companies and consumers have no motivation to encourage them to actively participate because of opportunistic behaviors, so they will choose the passive participation strategy.

**Fig 5:**
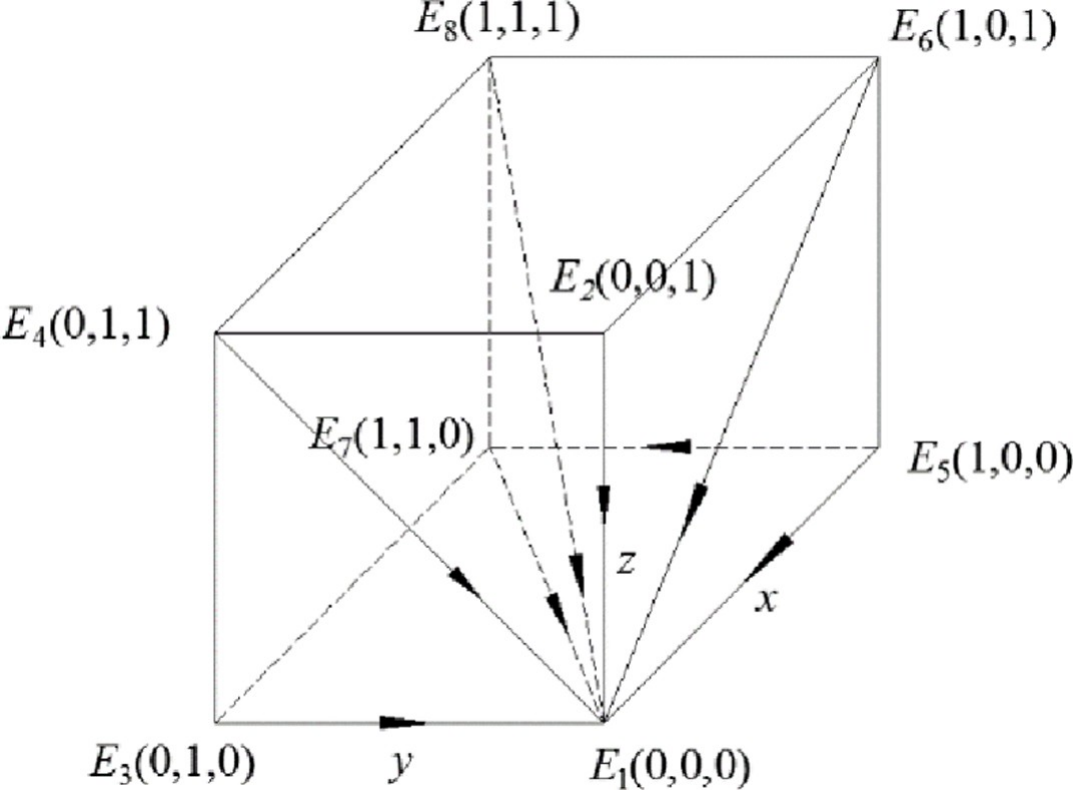
Phase diagram when the constraint condition is *R*_5_ –*R*_6_ < *C*_5_ –*C*_6_ + *B*_1_ + *B*_2_ –*F*_1_.

**Fig 6:**
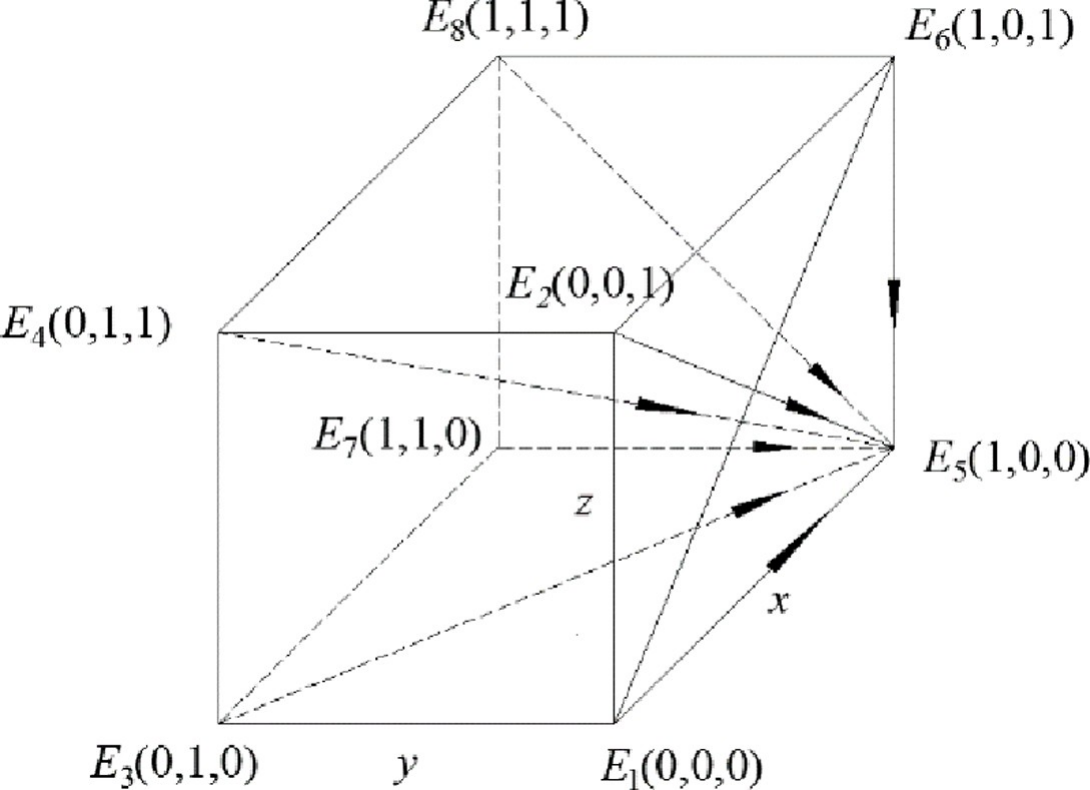
Phase diagram when the constraint condition is *R*_5_ –*R*_6_ > *C*_5_ –*C*_6_ + *B*_1_ + *B*_2_ –*F*_1_.

The phase diagrams of this model are shown in Figs 5 and 6.

## 6. Numerical simulation

The above assumptions and analysis are summarized, and the following variable constraints are obtained.


$$\left\{\begin{array}{c} C_{1}\geq C_{3},C_{2}\geq C_{4},C_{5}\geq C_{6}\\ R_{1}-C_{1}\leq R_{3}-C_{3},R_{2}-C_{2}\leq R_{4}-C_{4} \end{array}\right.$$

Since there is no specific national data, the following simulation selects a set of data that meets the criteria according to variable constraints. At this time, we assume that:

$$\begin{array}{l} A_{1}=5,A_{2}=4,F_{1}=3,C_{1}=5,C_{2}=4,\\ C_{3}=4,C_{4}=3,C_{5}=5,C_{6}=4,M_{1}=3,\\ M_{2}=1,M_{3}=2,M_{4}=1,B_{1}=3,B_{2}=2,\\ R_{1}=10,R_{2}=6,R_{3}=12,R_{4}=8 \end{array}$$

Based on the assumptions of the above parameters and the resulting replication dynamic equations, Matlab is used to simulate and analyze the evolutionary stabilization strategies of the government, waste drug recycling companies and consumers, and the evolutionary simulation results of the key components are discussed separately according to the relative returns of the government.

### 6.1. When the constraint is just *R*_5_ –*R*_6_ > *C*_5_ –*C*_6_ + *B*_1_ + *B*_2_ + *A*_1_ + *A*_2_

**Fig 7:**
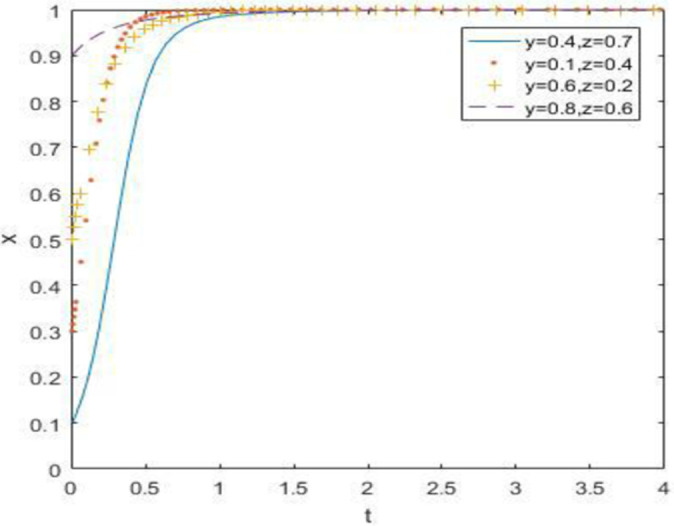
Simulation of government strategy evolution at *R*_5_ –*R*_6_ > *C*_5_ –*C*_6_ + *B*_1_ + *B*_2_ + *A*_1_ + *A*_2_.

**Fig 8:**
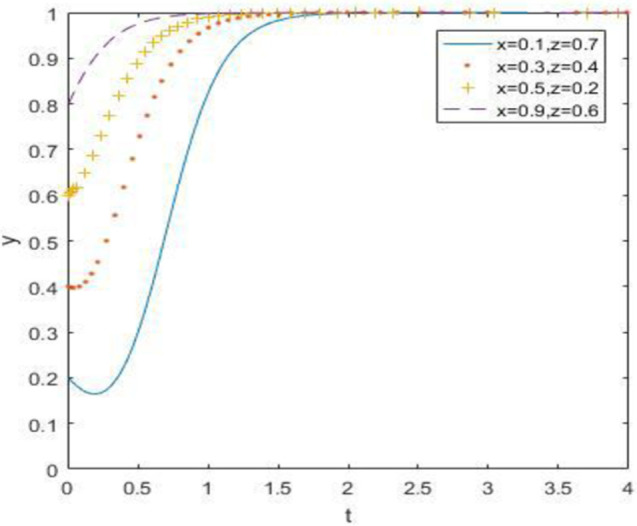
Strategy evolution simulation of discarded drug recycling companies at *R*_5_ –*R*_6_ > *C*_5_ –*C*_6_ + *B*_1_ + *B*_2_ + *A*_1_ + *A*_2_.

**Fig 9:**
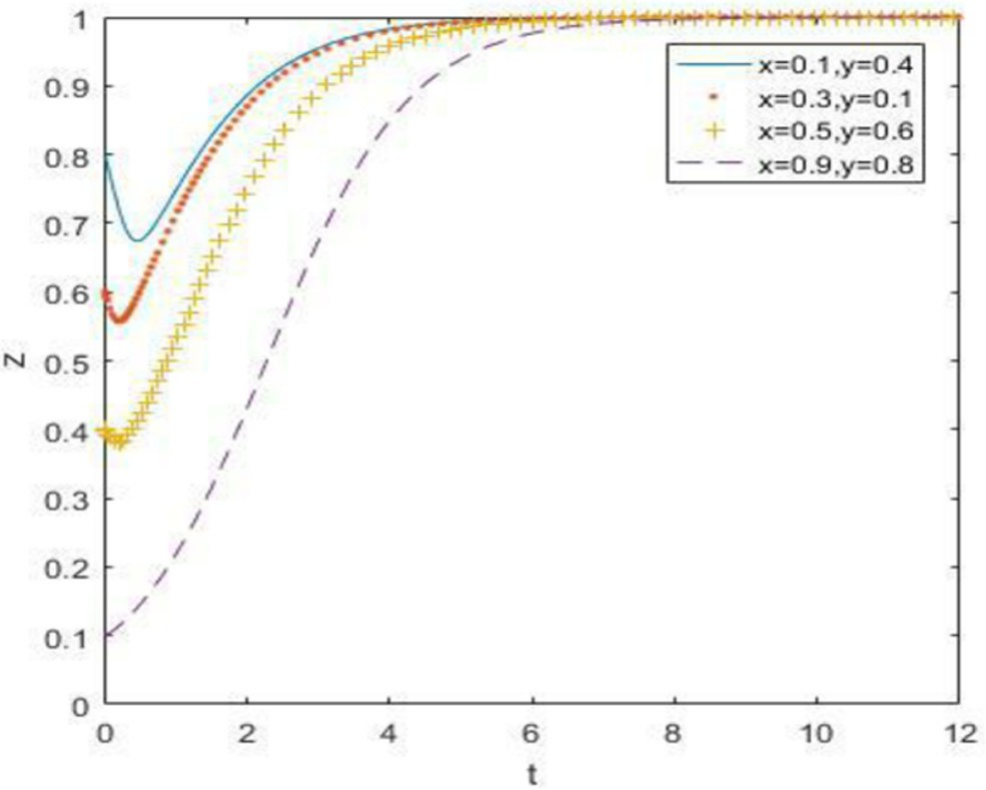
Evolution simulation of consumer strategy at *R*_5_ –*R*_6_ > *C*_5_ –*C*_6_ + *B*_1_ + *B*_2_ + *A*_1_ + *A*_2_.

At this point, we define *R*_5_ = 30, *R*_6_ = 14, and the simulation result is:

The evolutionary simulation results from Figs 7–9 show the evolution of the strategies of the government, waste drug recycling companies, and consumers when the relative gain to the government is high. The government chooses a strong regulatory strategy because of its relatively high gain, and under the influence of the strong regulatory strategy, both the recycling companies and consumers of discarded drugs take an active participation strategy. In this case, the evolutionary stability point is located at (1, 1, 1).

### 6.2. When the constraint is *R*_1_ –*C*_1_ ≥ *R*_3_ –*C*_3_, *R*_2_ –*C*_2_ ≥ *R*_4_ – *C*_4_ and *R*_5_ –*R*_6_ < *C*_5_ –*C*_6_ + *B*_1_ + *B*_2_ + *A*_1_ + *A*_2_

**Fig 10:**
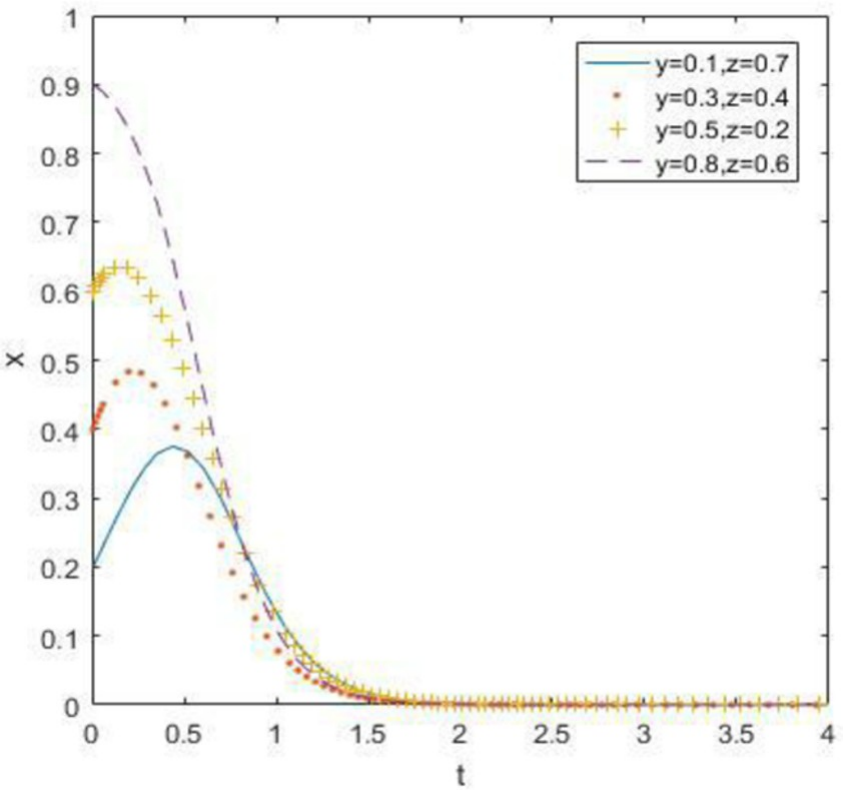
Simulation of government strategy evolution when *R*_1_ –*C*_1_ ≥ *R*_3_ –*C*_3_, *R*_2_ –*C*_2_ ≥ *R*_4_ –*C*_4_ and *R*_5_ –*R*_6_ < *C*_5_ –*C*_6_ + *B*_1_ + *B*_2_ + *A*_1_ + *A*_2_.

**Fig 11:**
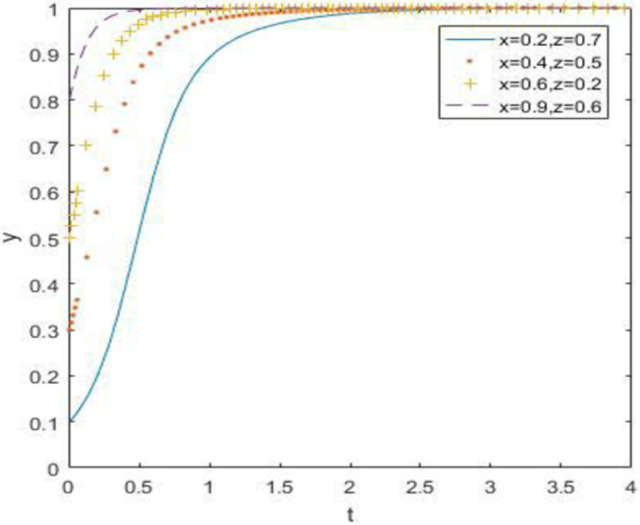
Strategy evolution simulation of discarded drug recycling companies when *R*_1_ –*C*_1_ ≥ *R*_3_ –*C*_3_, *R*_2_ –*C*_2_ ≥ *R*_4_ –*C*_4_ and *R*_5_ –*R*_6_ < *C*_5_ –*C*_6_ + *B*_1_ + *B*_2_ + *A*_1_ + *A*_2_.

**Fig 12:**
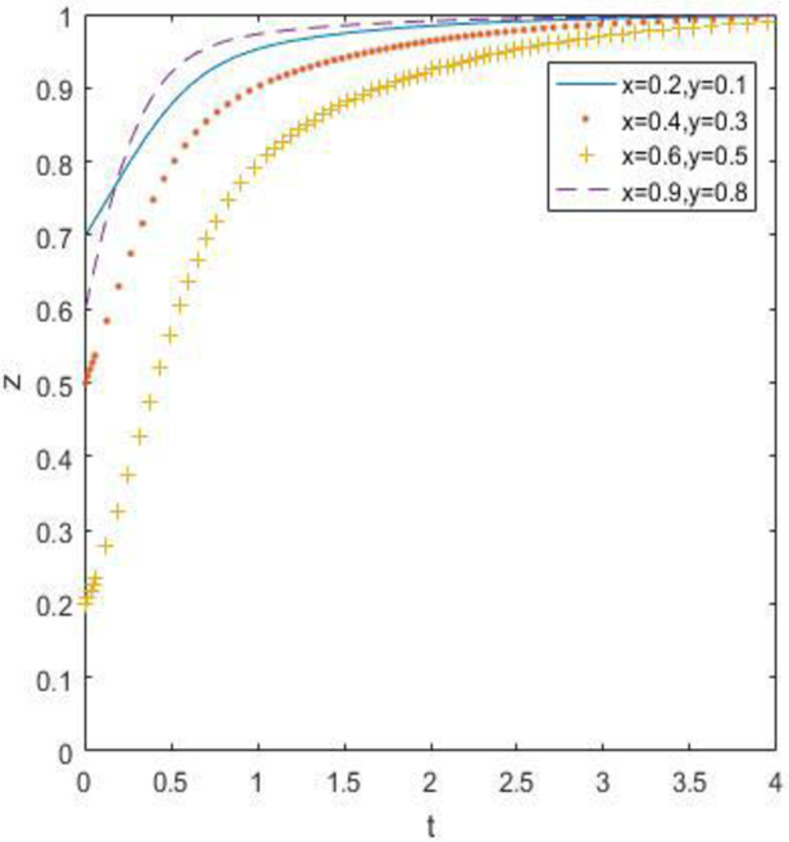
Simulation of consumer strategy evolution when *R*_1_ –*C*_1_ ≥ *R*_3_ –*C*_3_, *R*_2_ –*C*_2_ ≥ *R*_4_ –*C*_4_ and *R*_5_ –*R*_6_ < *C*_5_ –*C*_6_ + *B*_1_ + *B*_2_ + *A*_1_ + *A*_2_.

At this point, we define *R*_1_ = 15, *R*_2_ = 10, *R*_5_ = 30, *R*_6_ = 20, and the simulation result is:

The evolutionary simulation results from Figs 10–12 show that when the opportunity cost of enterprises and consumers is high, waste drug recycling enterprises and consumers will surely choose active participation behavior steadily after a long-term evolutionary process, regardless of whether the government chooses strong or weak regulation. Therefore, when the relative gain of the government is low, the evolutionary stability point is located at (0, 1, 1).

### 6.3. When the constraint is *R*_3_ –*C*_3_ –*F*_1_ > *R*_1_ –*C*_1_ + *A*_1_, *R*_4_ –*C*_4_ > *R*_2_ –*C*_2_ + *A*_2_ and *R*_5_ –*R*_6_ > *C*_5_ –*C*_6_ + *B*_1_ + *B*_2_ + *A*_1_ + *A*_2_

**Fig 13:**
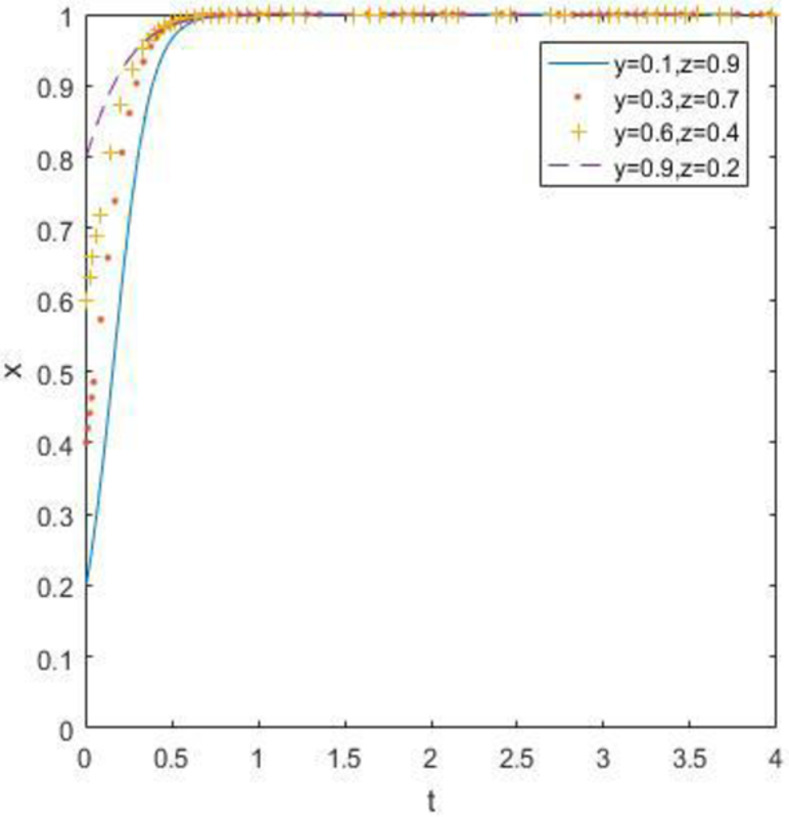
Simulation of government strategy evolution when *R*_3_ –*C*_3_ –*F*_1_ > *R*_1_ –*C*_1_ + *A*_1_, *R*_4_ –*C*_4_ > *R*_2_ –*C*_2_ + *A*_2_ and *R*_5_ –*R*_6_ > *C*_5_ –*C*_6_ + *B*_1_ + *B*_2_ + *A*_1_ + *A*_2_.

**Fig 14:**
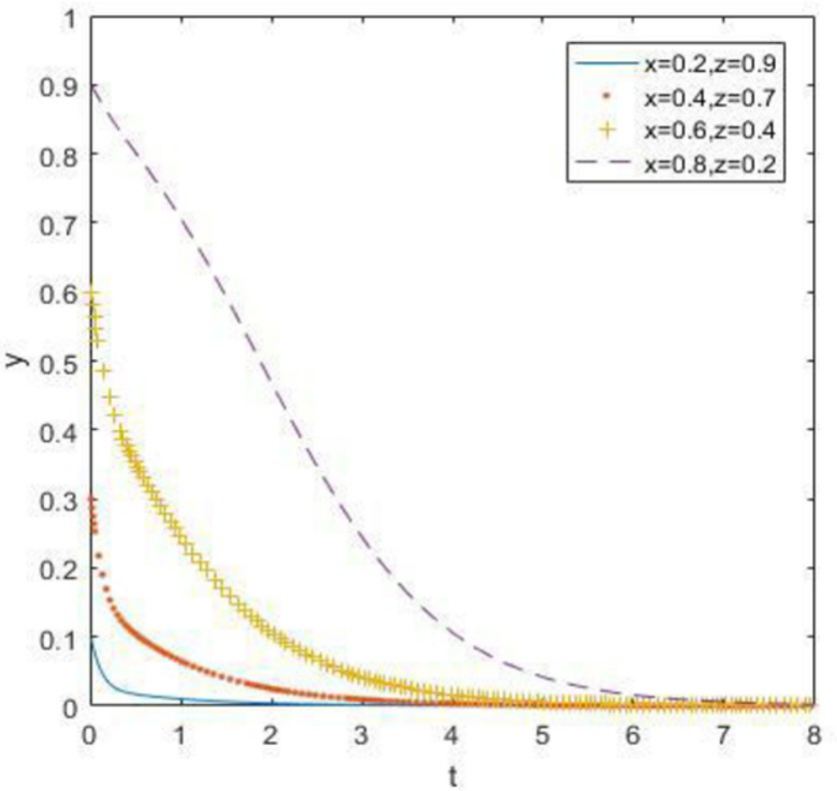
Strategy evolution simulation of discarded drug recycling companies when *R*_3_ –*C*_3_ –*F*_1_ > *R*_1_ –*C*_1_ + *A*_1_, *R*_4_ –*C*_4_ > *R*_2_ –*C*_2_ + *A*_2_ and *R*_5_ –*R*_6_ > *C*_5_ –*C*_6_ + *B*_1_ + *B*_2_ + *A*_1_ + *A*_2_.

**Fig 15:**
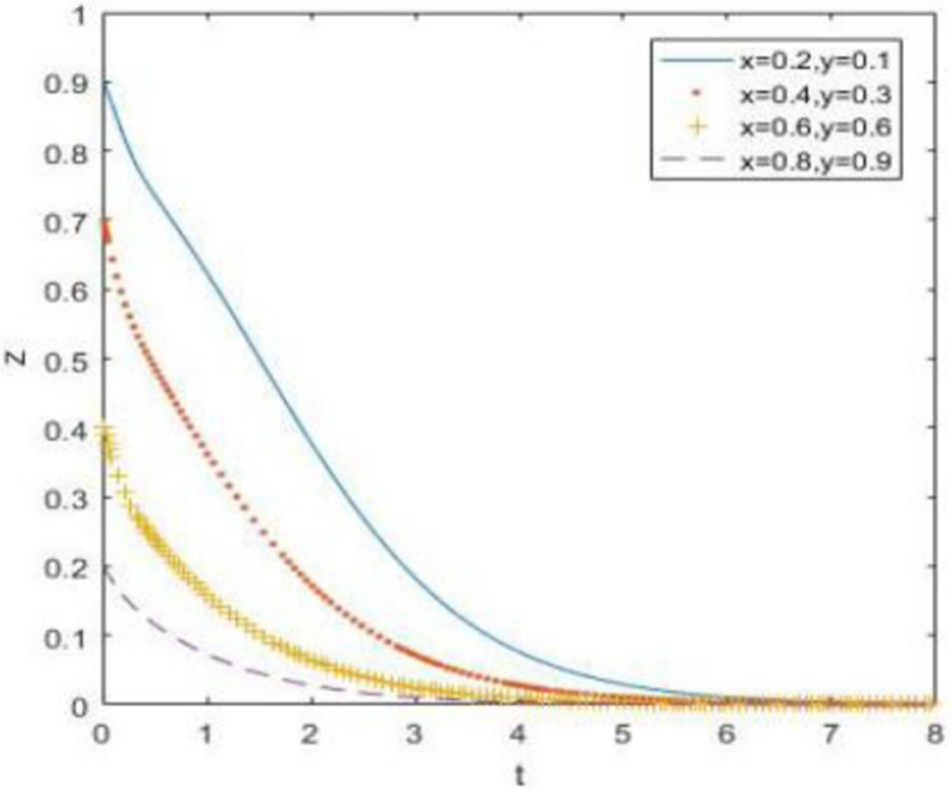
Simulation of consumer strategy evolution when *R*_3_ –*C*_3_ –*F*_1_ > *R*_1_ –*C*_1_ + *A*_1_, *R*_4_ –*C*_4_ > *R*_2_ –*C*_2_ + *A*_2_ and *R*_5_ –*R*_6_ > *C*_5_ –*C*_6_ + *B*_1_ + *B*_2_ + *A*_1_ + *A*_2_.

At this point, we define *R*_3_ = 18. *R*_4_ = 10, *R*_5_ = 30, *R*_6_ = 14, and the simulation result is:

According to the evolution simulation results in Figs 13–15, when the government supervision is still not strong enough, discarded drug recycling companies and consumers will stably jointly choose the recycling strategy of negative participation after a long period of evolution. When the relative gain of government regulation is relatively high, the government regulation enthusiasm is high. The only stable strategy combination of the system is (strong regulation, negative participation, negative participation), in other words, the evolutionary stability point is located at (1, 0, 0).

## 7. Conclusions

Discarded drugs have great harm, and the recycling mechanism of discarded drugs in China is not perfect, so the government must play a decisive role in the recycling process of discarded drugs. The government chooses strongly/weakly regulated companies and consumers out of consideration of its own interests. Recycling companies and consumers choose active/negative participation behaviors based on their own interests [44]. The above three subjects all affect the healthy development of the discarded drug recycling market. So, this paper considers a drug green recycling supply chain consisting of the government, drug recycling companies and consumers, describes the process of evolutionary game, carries out numerical analysis, and obtains the influence of relevant factors on the trend of evolutionary stability strategy. Finally, the strategy selection of the three main parties is clarified after the above analysis.

Some distinguished contributions of this study are as follows:

1. When the government’s relative income is relatively high, after a long-term strategy evolution, the government will choose a strong supervision strategy; otherwise, the government will choose a weak supervision strategy. And only when the government adopts a strong supervision strategy, can it change the opportunistic behavior of recycling companies and consumers, and enable them to actively participate in the recycling of discarded drugs.
2. Opportunistic costs affect the behavioral choices of waste drug recycling companies and consumers. When the opportunistic cost is low and the government chooses strong supervision, both recycling companies and consumers will actively participate in the recycling of discarded drugs; when the opportunistic cost is too high, regardless of the measures taken by the government, recycling companies and consumers will actively participate in discarded drugs Recycle.

we propose the following policy recommendations based on the above conclusions;

1. The government plays a leading role in the recycling process of discarded medicines from the perspective of the green supply chain. On the one hand, the government should continue to improve the laws and regulations related to drug recycling to solve the current problems of imperfect laws and regulations; on the other hand, the government should continue to increase supervision and maximize supervision [45–47]. The government should implement policy support, technical support, equipment investment and other measures for discarded drug recycling companies and consumers, and at the same time treat the recycling of discarded drugs with the strictest supervision [26, 27], in order to reduce the harm caused by discarded drugs. It can play a significant positive guiding role in the entire discarded drug recycling market, and promote the continuous progress of the ecological environment.
2. Discarded drug recycling companies should also actively participate in the recycling of discarded drugs, take effective measures to establish a good corporate image, and adopt various welfare measures to attract consumers to actively participate in the recycling of discarded drugs, and cooperate with the government to complete the recycling of discarded drugs and to win good income and image.
3. Consumers should also actively improve their own strength, pay attention to the cultivation of environmental protection awareness, and consciously develop the awareness of recycling of discarded drugs. Active participation makes the cooperation relationship with the enterprise stronger, and the excess income after the cooperation can be increased, thereby increasing its own income.

The joint efforts of the three main bodies are conducive to ensuring the healthy development of waste drug recycling market and promoting the continuous improvement of ecological environment.

Different from previous studies on single supply chain management risk, single supplier in supply chain or sustainable green production system, this paper combines supply chain and sustainable green ecosystem to analyze, and uses evolutionary game method to change the previous static and single analysis method. In the choice of the main body of the game, this paper chooses three main body of the game, the government, discarded drug recycling enterprises and consumers. This method changes the previous static single analysis method, and makes the theoretical analysis more in line with the actual situation of the discarded drug recovery market.

However, these three factors are not the only ones that constitute the whole recycling system, and the effects of other subjects or influencing factors are ignored in this paper. In this paper, only two strategies are provided for the subject. In future research, we can consider the evolutionary game of multi-agent and multi-strategy interactions, introduce a competition mechanism, consider the impact of multiple recycling companies on the recycling of discarded drug, and establish a multi-party game model, which will become the next research direction.
